# Factors Associated with Discharge Destination in Patients with Bone Metastases

**DOI:** 10.3390/medicina60060881

**Published:** 2024-05-27

**Authors:** Hanako Himematsu, Yukiyo Shimizu, Tami Yuhara, Kenta Hiasa, Masashi Yamazaki, Yasushi Hada

**Affiliations:** 1 Department of Rehabilitation Medicine, University of Tsukuba hospital, Tsukuba 305-8576, Japan; ariizumi.hanako.hs@ms.hosp.tsukuba.ac.jp (H.H.); yuhara_tami@outlook.jp (T.Y.); hiasa.kenta.ge@ms.hosp.tsukuba.ac.jp (K.H.); 2 Doctoral Program in Medical Sciences, Graduate School of Comprehensive Human Sciences, University of Tsukuba, Tsukuba 305-8575, Japan; 3 Department of Rehabilitation Medicine, Institute of Medicine, University of Tsukuba, Tsukuba 305-8575, Japan; y-hada@md.tsukuba.ac.jp; 4 Department of Orthopedic Surgery, Institute of Medicine, University of Tsukuba, Tsukuba 305-8575, Japan; masashiy@md.tsukuba.ac.jp

**Keywords:** neoplasm, bone metastasis, discharge destination, skeletal-related events

## Abstract

*Background and Objectives*: The discharge destination of patients with advanced cancer correlates with their quality of life. Patients with bone metastases often undergo lifestyle changes owing to pain and activity limitations. However, there are few reports on factors related to the discharge destination of patients with bone metastases. This study aimed to elucidate the factors associated with the discharge destination of patients with bone metastases. *Methods*: This study included 278 patients diagnosed with bone metastases who were admitted to the University of Tsukuba Hospital between April 2015 and March 2020. This study examined discharge destination, occurrence of skeletal-related events (SREs), primary lesions, locations of bone metastases, functional ambulation categories (FAC), age, and length of hospital stay. A binomial logistic regression analysis was conducted to compare the home and non-home discharge groups. *Results*: Of the 278 patients, 142 were discharged to home, 89 were discharged to somewhere other than home (non-home), and 47 died. The discharge destination was associated with spinal cord compression (SCC) (odds ratio [OR] 3.37, 95% confidence interval [CI] 1.35–8.43), hypercalcemia (OR 6.84, 95% CI 1.09–42.76), and FAC at admission (OR 0.45, 95% CI 0.35–0.58). The admission FAC cut-off value for discharge to home was determined to be 1.5 (area under the curve [AUC] 0.79, sensitivity 77.5%, specificity 68.5%). *Conclusions*: Factors associated with discharge destination were identified. The walking ability required for discharge to home was FAC 1.5, meaning that the patient needed one person to assist in preventing falls when walking on level ground. A cut-off value for FAC on admission for predicting outcomes was identified, suggesting the importance of gait ability assessment on admission.

## 1. Introduction

Advances in medical technology, cancer diagnosis, surgery, and drugs, as well as cancer screening and multidisciplinary treatment, have improved the survival rates of cancer patients. Technological advances have resulted in not only an increase in the number of patients cured, but also an upsurge in the number of patients living with cancer. As cancer progresses, recurrence and metastasis to surrounding blood vessels, lymph nodes, bones, and distant organs occur. Bone is the third most common site of solid cancer metastasis, with 70% of prostate and breast cancer patients having bone metastasis [1]. With the improvement in the survival of cancer patients, the number of patients who develop bone metastases is expected to increase.

Patients with bone metastases are at risk of skeletal-related events (SREs), including pathological fractures, spinal cord compression (SCC), hypercalcemia, orthopedic surgical intervention, and palliative radiation directed at the bone [2], and may experience limitations in the activities of daily living (ADLs). Fractures of load-bearing bones, such as the spine, pelvis, and lower extremities, and SCC have a significant impact on the ADLs. This not only reduces the quality of life (QOL), but also makes it difficult to continue treatment of the primary tumor due to pain, decline in physical function, and hospitalization for bone metastasis treatment. The occurrence of SREs decreases the survival rate [3]; therefore, it is important to prevent the SREs as far as possible after the diagnosis of bone metastasis. Previous studies have elucidated the benefits of rehabilitation for patients with bone metastasis. Rehabilitation can improve the physical function of patients with bone metastases without fractures or bone pain [4]. Patients who moved to a rehabilitation hospital after surgical stabilization of their pathological fractures experienced a notable improvement of their mobility and ADLs [5]. In another study, the functional outcomes of patients with metastatic SCC significantly improved through a rehabilitation program [6]. However, rehabilitation for patients with bone metastasis carries the risk of new SREs, such as pathological fractures and the appearance or exacerbation of SCC symptoms. Nevertheless, the advantages of physical therapy are considered greater than the disadvantages [7]. Since April 2018, our hospital has been conducting a bone metastasis liaison conference to enhance the exchange of information among healthcare professionals regarding patients with bone metastases. Orthopedic surgeons, rehabilitation physicians, radiation oncologists, primary physicians, physical therapists, and occupational therapists participate in this conference. At this conference, the status of the underlying disease and treatment details including chemotherapy, radiation therapy, and palliative treatment, are shared. Furthermore, radiological findings are discussed, and appropriate rehabilitation intervention methods are discussed based on bone strength and rest. Therapist feedback includes the patient’s ADL status, pain levels, and pain exacerbating factors, which help determine the treatment plan. Based on the results of the conference, rehabilitation intervention is provided according to the stage of the disease, including instruction on movement methods and exercise intervention to reduce the burden on bone metastases.

The discharge destination is important for patients with advanced cancer for their QOL, and prognosis-based rehabilitation and care enables a shorter length of hospital stay. Furthermore, home-based supportive care improves the social and emotional QOL of patients with advanced cancer [8]. In stroke patients, a systematic review and meta-analysis reported that higher independence in ADLs on admission predicted a return to home [9]. Regarding cancer patients, performance status, age [10], and frailty [11] have been reported as factors associated with discharge home, but no definite conclusion has been reached. In addition, few studies have examined the factors associated with the discharge of patients with bone metastases [12,13].

This study aimed to identify the factors associated with the discharge destination of patients with bone metastasis.

## 2. Materials and Methods

### 2.1. Design and Participants

This was a single-center, retrospective, observational study. Patients with cancer admitted to University of Tsukuba Hospital between April 2015 and March 2020 were included in this study. Patients were selected based on the diagnosis procedure combination (DPC) indicating “cancer”, “carcinoma”, “tumor”, “sarcoma”, or “metastatic bone tumor”. The eligibility criterion was diagnosis with bone metastasis. Patients were excluded if they had hematopoietic tumors, were under 18 years of age at diagnosis, had missing data, and had no history of hospitalization after the diagnosis of bone metastasis. Bone metastasis was defined as “bone tumor”, or “bone metastasis” in the imaging reports or medical records, or “bone metastasis” in the DPC. The date of diagnosis was determined to be the date on which “bone tumor” or “bone metastasis” was documented in the imaging reports or medical records.

### 2.2. Data Collection

Patient data extracted from the medical records included age, sex, primary tumor type, date of bone metastasis diagnosis, and bone metastasis site. For patients with a history of hospitalization after the diagnosis of bone metastasis, the following information was collected: SREs, including pathological fracture, SCC, hypercalcemia, radiation therapy, orthopedic surgery, as well as discharge destination, length of stay, history of bone-modifying agent use, orthopedic consultation, palliative care team consultation, with or without rehabilitation treatment, activity limitation, and ambulatory capacity at admission and discharge. Among the bone metastasis sites, the vertebral column, pelvis, including the hip and sacrum, and lower limb bones were defined as load-bearing bones. One hospitalization per patient was considered evaluable.

### 2.3. Ambulatory Capacity

Functional ambulatory capacity was assessed using functional ambulation categories (FAC). The FAC identify six levels of walking ability based on the amount of physical support required. A score of 0 indicates that the patient is a nonfunctional ambulator (cannot walk); a score of 1, 2, or 3 denotes a dependent ambulator who requires assistance from another person in the form of continuous manual contact (1), continuous or intermittent manual contact (2), or verbal supervision/guarding (3); and a score of 4 or 5 describes an independent ambulator who can walk freely on level surfaces only (4) or any surface (5 = maximum score) [14].

### 2.4. Statistical Analysis

The Mann–Whitney U test was used to compare basic subject information, SREs, and FAC between the two groups, and one-way analysis of variance was used to compare data among the three groups. Binary logistic regression analyses were conducted to examine factors associated with the admission and discharge FAC. The admission or discharge FAC (0 for the low category with scores of 0–2 and 1 for the high category with scores of 3–5) was designated as the dependent variable. The independent variables for the admission FAC analysis were sex, age at admission, number of admissions, metastasis to load-bearing bones, SREs, and activity limitations. Those for the discharge FAC analysis were sex, age at admission, number of admissions, load-bearing bone metastases, and SREs. Spearman’s rank correlation analysis was performed to investigate the association between the admission and discharge FAC. Binary logistic regression analysis was performed to clarify the factors associated with the discharge destination. The discharge outcome (excluding death) was designated as the dependent variable, whereas the independent variables were sex, age at admission, days from diagnosis, length of hospital stay, load-bearing bone metastasis, admission FAC, SREs, and rehabilitation. Receiver operating characteristic (ROC) curve analysis was performed on the variables selected in the binary logistic regression analysis to calculate the area under the curve (AUC) and determine the cut-off value. Discrimination performance was evaluated by calculating sensitivity and specificity. The cut-off point was determined as the point on the ROC curve with the minimum distance from the top-left corner. IBM SPSS Statistics ver27.0.1.0 (IBM Corporation, Armonk, NY, USA) was used for all analyses, and the significance level was set at 5%. This study was approved by the Ethical Review Committee for Clinical Research at the University of Tsukuba Hospital (R02-033). Patient consent was exempted because data from past medical records were anonymized, and an opt-out option was provided on the website.

## 3. Results

### 3.1. Patient Characteristics

**Figure 1:**
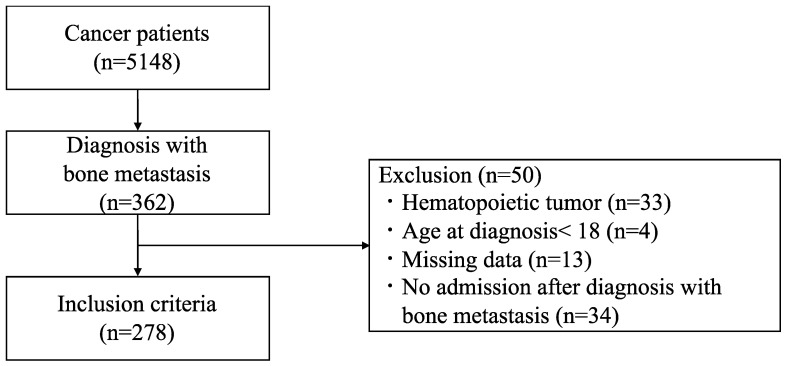
Patient flow diagram.

Of the 5148 patients with cancer, 362 (7.0%) were diagnosed with bone metastasis. Eighty-four patients were excluded for the following reasons: 33 had hematopoietic tumors, 4 were under 18 years of age, 34 were not admitted after the diagnosis of bone metastasis, and 13 had missing data. A total of 278 patients were included in the analysis. Of these patients, 142 were discharged to home, 84 to other hospitals, 5 to nursing homes, and 47 died at our hospital (Figure 1). Table 1 shows the patient characteristics. There were no significant differences in sex, primary tumor, and history of SREs among the groups. Table 2 shows the hospitalization status, and Table 3 shows the site of pathological fracture. There were no significant differences in age, days from the diagnosis of bone metastasis, or hospitalization period by discharge destination. Load-bearing bone metastasis occurred in over 90% of each group. In terms of SREs, the non-home group had a significantly higher incidence of SCC than the home group (*p* < 0.001). Both the home and non-home groups had a significantly higher rate of orthopedic surgery than the deceased group (*p* = 0.014, 0.004). No significant differences were observed in pathological fractures, hypercalcemia, or radiotherapy. Also, no significant differences were observed in the site of pathological fracture and rate of rehabilitation intervention. The rehabilitation program included strength training, joint range of motion exercises, walking exercises, ADL training, and palliative interventions. No new SREs were reported throughout the rehabilitation interventions. Postoperative orthopedic patients engaged in consultations with orthopedic surgeons during rehabilitation to discuss the need for trunk or limb orthoses, and to assess the appropriate load and level of activity. The activity levels and treatment strategies for other patients were discussed at the bone metastasis liaison conference, which was attended by orthopedic surgeons, radiologists, rehabilitation physicians, and physical therapists.

### 3.2. Admission and Discharge FAC

**Figure 2:**
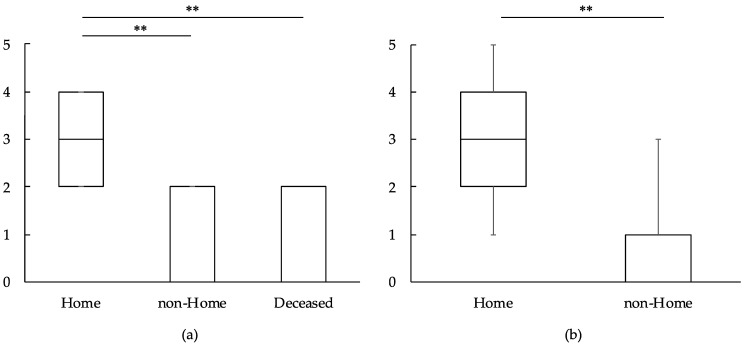
(**a**) Admission FAC: The admission FAC for the home group was significantly higher than that for the non-home and deceased groups. (**b**) Discharge FAC: The discharge FAC for the home group was significantly higher than that for the non-home group. FAC: Functional ambulation categories. **: *p* < 0.01.

Admission FAC was significantly higher in the home group than in the non-home and deceased groups (*p* < 0.001) (Figure 2a). Discharge FAC was significantly higher in the home group than in the non-home group (*p* < 0.001) (Figure 2b). In the admission FAC, the adjusted odds ratio (OR) for load-bearing bone metastasis was 0.161 (95% confidence interval [CI]: 0.042–0.614, *p* = 0.008) (Table 4). Discharge FAC was associated with age and load-bearing bone metastasis. The adjusted ORs were 0.966 (95% CI: 0.942–0.992, *p* = 0.009) and 0.160 (95% CI: 0.040–0.640, *p* = 0.010), respectively (Table 5). There was a significantly strong correlation between admission and discharge FAC (r = 0.780, *p* < 0.001).

### 3.3. Discharge Destination

**Figure 3:**
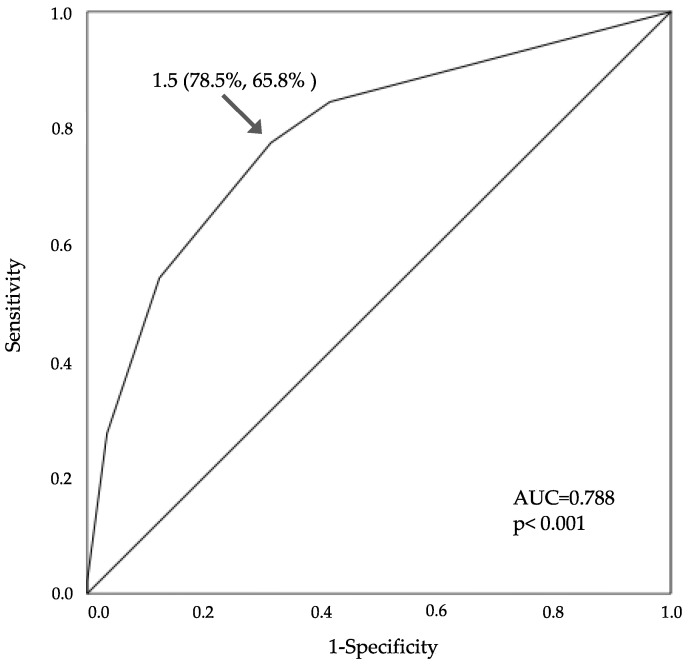
ROC curve of admission FAC for predicting discharge destination. The cut-off value of admission FAC for discharge to home was 1.5 (AUC 0.788; 95% CI: 0.729–0.848, sensitivity 78.5%, specificity 65.8%, *p* < 0.001). ROC: receiver operating characteristic, FAC: functional ambulation categories, AUC: are under the curve, CI: confidence interval.

The discharge destination was associated with SCC, hypercalcemia, and admission FAC. The adjusted OR for SCC was 3.373 (95% CI: 1.345–8.462, *p* = 0.010), for hypercalcemia was 6.835 (95% CI: 1.092–42.758, *p* = 0.040), and for admission FAC was 0.451 (95% CI: 0.353–0.576, *p* < 0.001) (Table 6). The cut-off value for admission FAC was 1.5 (AUC, 0.788; 95% CI: 0.729–0.848, sensitivity 77.5%, specificity 68.5%, *p* < 0.001) (Figure 3).

## 4. Discussion

This study examined the factors associated with the discharge destination of patients with bone metastases. The main finding was that the discharge destination of these patients was associated with SCC, hypercalcemia, and FAC at admission. In addition, the cut-off value for FAC at admission was 1.5.

In the present study, bone metastasis occurred in 7.0% of all patients, which concurs with the results of previous studies showing that 7.5–8.6% of patients with advanced cancer develop bone metastasis [15,16,17]. Furthermore, a major proportion of primary cancers comprised prostate, lung, breast, and kidney cancers, which is in accordance with the reported cancer types with a high risk of developing bone metastasis [15,16,17]. Therefore, the individuals included in this study can be considered representative of the general population. Approximately 60% of patients had SREs at admission, an observation similar to those in previous studies in which the incidence of SREs varied from 48–69% [18].

Many studies have shown that ADL on admission and discharge were predictors of discharge destination of stroke patients [9,19,20]. The discharge destination predictors have been reported for several diseases, including spinal cord injury and post-proximal femoral fracture [21,22]. Regarding bone metastasis, a previous retrospective observational study reported that the discharge destination was associated with discharge ADL, and the cut-off value of the Barthel Index was 72.5 [13]. Another study showed that discharge to home was associated with the Barthel Index at rehabilitation initiation, with a cut-off value of 60 [12]. A Barthel Index score of 21–60 indicates “severe” dependency and 61–90 indicates “moderate” dependency [23]. In the present study, the cut-off value of FAC for home discharge was 1.5, indicating a moderate dependency on walking. Therefore, the results of this study are consistent with previous reports.

Our study results indicated that walking ability at admission was significantly higher in the home group than in the non-home or deceased group. Logistic regression analysis identified metastasis to load-bearing bones as a factor related to walking ability at admission. Coleman et al. suggested that load-bearing bone metastases (i.e., vertebral, pelvic, and femoral lesions) cause bone pain [18]. Previous research also found that two-thirds of patients with bone metastasis experienced severe pain, and that loading pain impaired walking [24]. Furthermore, cancer progression causes a decline in physical and mental function, limiting activities and walking ability. Older cancer patients, specifically, are reported to be more prone to a decline in physical abilities, such as grip strength and walking speed [25,26]. Many patients with cancer have cachexia and metabolic wasting syndrome, which is characterized by unintended weight loss, anorexia, and a skeletal muscle catabolic state. Cachexia can cause muscle weakness, reduced physical function, and higher mortality [27,28]. In addition, chemotherapy and radiation therapy are known to reduce bone mineral density [29], cause loss of lean body mass, and weaken muscles [28,30]. Sarcopenia, age-related muscle loss, reduces cardiopulmonary function and physical activity in patients with cancer [31]. Thus, systemic inflammatory conditions, age-related decline in physical function, and reduced activity due to treatment-related muscle weakness and osteoporosis may influence the decline in walking ability of patients with cancer.

Similarly, walking ability at discharge was significantly higher in the home group than in the non-home group. The logistic regression analysis results showed that age and metastasis to load-bearing bones were associated with walking ability at discharge. Frailty is defined as a state of increased vulnerability due to multiple age-related physiological declines in function and reserve [32], and approximately 10% of older adults aged over 65 years and 25–50% of those aged over 85 years become frail after the onset of stressors, including cancer treatment [33]. In addition, ADL decline is 1.6–2.0 times greater and mortality is 1.8–2.3 times greater in frail patients than in healthy individuals [34], with a significant impact on prognosis. Cancer patients, with a low reserve capacity, are more likely to become frail when hospitalized, as they are less motivated to be active due to treatment or primary illness and are compelled to rest excessively, which may influence the decline in walking ability. In addition, rest has been reported to cause muscle weakness of 1–3% per day [35]. Thus, activity limitation may lower mobility, and walking ability is reduced due to disuse during the rest period, even after activity limitation is lifted.

The findings of this study demonstrate that SCC and hypercalcemia are associated with discharge destination. Over 95% of SCC patients have back pain [36], and the feeling of weakness, the second most common symptom, is strongly correlated with walking ability, with 50–68% having difficulty walking at diagnosis. Other symptoms include sensory disturbance in 50–70% and bladder-rectal disturbance in 50–60%, with bladder-sphincter disturbance being a poor prognostic factor for walking ability [36,37]. Although there was no association between SCC and walking ability in this study, physical symptoms related to bone metastasis, such as back pain, sensory disturbances, and bladder-rectal impairment, may have influenced the outcomes. Hypercalcemia also occurs in 20–30% of patients with cancer and causes renal failure and progressive mental disorders, including coma [38]. Cancer-related hypercalcemia is associated with disseminated disease and has a poor prognosis, with a median survival of 3–4 months [39], and a mortality rate of approximately 50% within 30 days [38]. This suggests that hypercalcemia may reduce activity and affect patient outcomes.

Our study found no difference in the rate of rehabilitation intervention by discharge destinations. Rehabilitation for patients with bone metastasis aims to protect patients from being bedridden and to maintain ADLs for as long as possible, but it carries the risk of new SREs such as pathological fracture and the appearance or exacerbation of SCC symptoms. However, in a previous study, the incidence of pathologic fractures of long bones during physical therapy was not very high, and the advantages of physical therapy outweighed the disadvantages. In addition, the risk of bed rest without rehabilitation was greater than the risk of many complications from disuse due to bed rest [7]. Therefore, discussing the risks and benefits of rehabilitation intervention in a multidisciplinary team [18] can improve the patients’ ADL and QOL. Multidisciplinary approach also ensures optimal diagnosis and treatment. Accurate diagnosis and appropriate therapeutic interventions for bone metastases require a multifaceted array of evaluations and specialist input from attending physicians, radiation oncologists, orthopedic surgeons, rehabilitation physicians, pathologists, clinical oncologists, palliative care physicians, physical therapists, occupational therapists, and other healthcare professionals [40]. Implementing a multidisciplinary collaborative team model can provide comprehensive physical and psychological care to patients [41], leading to improved quality of life and a more favorable prognosis. Furthermore, it facilitates the initiation and maintenance of systemic treatment regimens targeting the primary tumor [40].

This study had a few limitations. First, the population under investigation had a diverse range of cancer types. Second, there was variation in the time since the diagnosis of bone metastasis. Variations in cancer type contribute to disparities in the occurrence of bone metastasis types and SREs. The bone metastasis type may have different effects on bone strength and fracture susceptibility. Third, the rehabilitation content, such as exercise content and load, was not standardized and depended on the discretion of the therapist. Finally, there was a lack of information on the home environment, lifestyle, social factors (such as marital status, number of children), and family caregiving ability, as factors affecting outcomes.

However, to the best of our knowledge, this is the first study to clarify the cut-off value for the gait ability of patients with bone metastasis to discharge to home. The results of this study may be useful in determining the discharge destination of patients with bone metastasis. In future, based on this study, we would like to determine the relationship between patient circumstances (i.e., home environment, lifestyle, and family structure) and discharge destination.

## 5. Conclusions

Our study identified factors associated with discharge destination, which were FAC at admission, SCC, and hypercalcemia. The FAC at admission required for discharge to home was higher than 1.5, meaning that the patient needed one person to assist in preventing falls when walking on level ground. Our study highlights the importance of assessing the walking ability of patients with bone metastases at admission for multidisciplinary collaboration at an early stage. Future studies should examine the relationship between the patients’ personal circumstances and discharge destination.

## Figures and Tables

**Table 1:** Patient characteristics.

Discharge Destination	All *n* = 278	Home *n* = 142	Non-Home *n* = 89		Deceased *n* = 47	*p*-Value
			Other Hospital *n* = 84	Nursing Facility *n* = 5		
Sex						
Male	131 [47.1]	67 [47.2]	41 [48.8]	1 [20.0]	22 [46.8]	0.667
Female	147 [52.9]	75 [52.8]	43 [51.2]	4 [80.0]	25 [53.2]	
Primary tumor						
Lung	65 [23.4]	34 [23.9]	16 [19.0]		15 [31.9]	
Breast	60 [21.6]	28 [19.7]	21 [25.0]	4 [80.0]	7 [14.9]	
Kidney	20 [7.2]	10 [7.0]	8 [9.5]		2 [4.3]	
Uterine	18 [6.5]	8 [5.6]	5 [6.0]		5 [10.6]	
Prostate	16 [5.8]	14 [9.9]	2 [2.4]			
Colon	13 [4.7]	7 [4.9]	3 [3.6]		3 [6.4]	
Liver	9 [3.2]	6 [4.2]	2 [2.4]		1 [2.1]	
Thyroid	9 [3.2]	4 [2.8]	4 [4.8]		1 [2.1]	
Skin	9 [3.2]	2 [1.4]	2 [2.4]		5 [10.6]	
Stomach	8 [2.9]	3 [2.1]	3 [3.6]		2 [4.3]	
Esophageal	7 [2.5]	4 [2.8]	3 [3.6]			
Soft Tissue Sarcomas	7 [2.5]	4 [2.8]	1 [1.2]		2 [4.3]	
Pancreatic	7 [2.5]	4 [2.8]	2 [2.4]		1 [2.1]	
Unknown	5 [1.8]	1 [0.7]	1 [1.2]		1 [2.1]	
Other	25 [9.5]	13 [9.1]	9 [10.8]	1 [20.0]	2 [4.2]	
History of SREs						
Pathological fracture	11 [4.0]	3 [2.1]	5 [6.0]	1 [20.0]	2 [4.3]	0.137
SCC	10 [3.6]	4 [2.8]	3 [3.6]	0	3 [6.4]	0.686
Hypercalcemia	0	0	0	0	0	1
Radiotherapy	46 [16.5]	23 [16.2]	15 [17.9]	2 [40.0]	6 [12.8]	0.46
Orthopedic surgery	7 [2.5]	3 [2.1]	4 [4.8]	0	0	0.369
One or more SREs	49 [17.6]	22 [15.5]	17 [20.2]	2 [40.0]	8 [17.0]	0.463

Values are presented as number [%]; SREs: skeletal-related events; SCC: spinal cord compression.

**Table 2:** Hospitalization status.

	Home	Non-Home	Deceased	*p*-Value
Age *	66.2 ± 11.3	66.4 ± 13.3	62.8 ± 13.2	0.24
Days from diagnosis *	267.6 ± 493.0	351.5 ± 540.8	154.8 ± 327.0	0.079
Hospitalization period *	36.1 ± 28.3	38.9 ± 24.1	34.2 ± 24.6	0.67
Load-bearing bone metastasis	130 [91.5]	86 [96.6]	45 [95.7]	0.247
SREs				
Pathological fracture	30 [21.1]	23 [25.8]	10 [21.3]	0.686
SCC	22 [15.5]	33 [37.1] ^§^	12 [25.5]	<0.001
Hypercalcemia	3 [2.1]	7 [7.9]	3 [6.4]	0.11
Radiation therapy	49 [34.5]	37 [41.6]	16 [34.0]	0.511
Orthopedic surgery	27 [19.0] ^†^	21 [23.6] ^†^	3 [6.4]	0.046
One or more SREs	83 [58.5]	56 [62.9]	27 [57.4]	0.751
Multidisciplinary intervention				
Orthopedics intervention	90 [63.4] ^†^	65 [73.0] ^†, §^	24 [51.1]	0.037
Activity limiting	31 [34.4]	22 [34.4]	14 [58.3]	0.872
Palliative care	44 [31.0]	49 [55.1] ^§^	30 [63.8] ^§^	<0.001
Rehabilitation	132 [93.0]	85 [95.5]	41 [87.2]	0.206

* Mean ± standard deviation, other values are presented as number [%], SREs: skeletal-related events, FAC: functional ambulation categories, SCC: spinal cord compression, ^†^: Indicates a significant difference from the home group, ^§^: Indicates a significant difference from the deceased group.

**Table 3:** Site of pathological fracture.

	Home	Non-Home	Deceased	*p*-Value
Pathological fracture				0.07
Spine	19 [63.3]	16 [69.6]	7 [70.0]	
Thigh	5 [16.7]	6 [26.1]	0	
Humerus	3 [10.0]	0	0	
Pelvis	3 [10.0]	0	3 [30.0]	
Others	0	1 [4.3]	0	

Values are presented as number [%].

**Table 4:** Odds ratios and 95% confidence intervals of admission FAC.

Explanatory Variable	OR	95% CI	*p*-Value
Age	0.989	0.964–1.014	0.367
Load-bearing bone metastasis	0.161	0.042–0.614	0.008
Sex	0.966	0.536–1.744	0.910
Pathological fracture	0.547	0.229–1.306	0.174
SCC	0.583	0.250–1.359	0.211
Hypercalcemia	0.318	0.052–1.929	0.213
Radiation therapy	0.879	0.330–2.339	0.796
Orthopedic surgery	1.240	0.414–3.717	0.701
SREs	1.179	0.409–3.401	0.760
Activity limiting	0.474	0.221–1.018	0.056
Times of admission	0.970	0.815–1.154	0.731

SREs: skeletal-related events, FAC: functional ambulation categories, SCC: spinal cord compression, OR: odds ratio, CI: confidence interval.

**Table 5:** Odds ratios and 95% confidence intervals of discharge FAC.

Explanatory Variable	OR	95% CI	*p*-Value
Age	0.970	0.946–0.995	0.018
Load-bearing bone metastasis	0.136	0.340–0.541	0.005
Sex	1.079	0.604–1.927	0.796
Pathological fracture	1.228	0.557–2.705	0.611
SCC	0.648	0.287–1.462	0.296
Hypercalcemia	0.240	0.042–1.376	0.109
Radiation therapy	0.668	0.264–1.685	0.392
Orthopedic surgery	0.756	0.269–2.122	0.595
SRE	1.036	0.372–2.881	0.946
Times of admission	0.964	0.812–1.145	0.679

SREs: Skeletal-related events, SCC: Spinal cord compression, OR: odds ratio, CI: confidence interval, FAC: functional ambulation categories.

**Table 6:** Discharge destination-related factors.

Explanatory Variable	OR	95% CI	*p*-Value
Age	1.007	0.979–1.036	0.626
Sex	0.903	0.449–1.815	0.775
Days from diagnosis	1.001	1.000–1.001	0.125
Admission days	1.001	0.988–1.015	0.840
Load-bearing bone metastasis	0.979	0.186–5.157	0.980
Admission FAC	0.451	0.353–0.576	<0.001
SRE	0.345	0.094–1.267	0.109
Pathological fracture	0.816	0.320–2.077	0.669
SCC	3.373	1.345–8.462	0.010
Hypercalcemia	6.835	1.092–42.758	0.040
Radiation therapy	1.878	0.600–5.872	0.279
Orthopedic surgery	1.625	0.448–5.894	0.460
Rehabilitation	1.184	0.245–5.725	0.834

SREs: skeletal-related events, FAC: functional ambulation categories, SCC: spinal cord compression, OR: odds ratio, CI: confidence interval.

## Data Availability

Data are contained within the article.
